# Isolation of Circulating Tumour Cells in Patients With Glioblastoma Using Spiral Microfluidic Technology – A Pilot Study

**DOI:** 10.3389/fonc.2021.681130

**Published:** 2021-06-03

**Authors:** Juliana Müller Bark, Arutha Kulasinghe, Gunter Hartel, Paul Leo, Majid Ebrahimi Warkiani, Rosalind L. Jeffree, Benjamin Chua, Bryan W. Day, Chamindie Punyadeera

**Affiliations:** ^1^ Saliva and Liquid Biopsy Translational Laboratory, School of Biomedical Sciences, Faculty of Health, Queensland University of Technology, Brisbane, QLD, Australia; ^2^ Translational Research Institute, Brisbane, QLD, Australia; ^3^ Department of Statistics, QIMR Berghofer Medical Research Institute, Brisbane, QLD, Australia; ^4^ Translational Genomics Group, School of Biomedical Sciences, Faculty of Health, Queensland University of Technology, Brisbane, QLD, Australia; ^5^ The School of Biomedical Engineering, University of Technology Sydney, Sydney, NSW, Australia; ^6^ Faculty of Medicine, University of Queensland, Brisbane, QLD, Australia; ^7^ Kenneth G. Jamieson Department of Neurosurgery, Royal Brisbane and Women’s Hospital, Brisbane, QLD, Australia; ^8^ Cell and Molecular Biology Department, Sid Faithfull Brain Cancer Laboratory, QIMR Berghofer MRI, Brisbane, QLD, Australia; ^9^ Cancer Care Services, Royal Brisbane and Women’s Hospital, Brisbane, QLD, Australia; ^10^ School of Biomedical Sciences, Faculty of Health, Queensland University of Technology, Brisbane, QLD, Australia

**Keywords:** glioblastoma, liquid biopsy, circulating tumour cells, glioma, spiral microfluidic technology

## Abstract

Glioblastoma (GBM) is the most common and aggressive type of tumour arising from the central nervous system. GBM remains an incurable disease despite advancement in therapies, with overall survival of approximately 15 months. Recent literature has highlighted that GBM releases tumoural content which crosses the blood-brain barrier (BBB) and is detected in patients’ blood, such as circulating tumour cells (CTCs). CTCs carry tumour information and have shown promise as prognostic and predictive biomarkers in different cancer types. Currently, there is limited data for the clinical utility of CTCs in GBM. Here, we report the use of spiral microfluidic technology to isolate CTCs from whole blood of newly diagnosed GBM patients before and after surgery, followed by characterization for GFAP, cell-surface vimentin protein expression and EGFR amplification. CTCs were found in 13 out of 20 patients (9/20 before surgery and 11/19 after surgery). Patients with CTC counts equal to 0 after surgery had a significantly longer recurrence-free survival (p=0.0370). This is the first investigation using the spiral microfluidics technology for the enrichment of CTCs from GBM patients and these results support the use of this technology to better understand the clinical value of CTCs in the management of GBM in future studies.

## Introduction

Glioblastoma (GBM) is the most frequent and aggressive type of brain cancer in adults (1). Despite the standard of care treatment (surgery followed by radio and chemotherapy), overall survival is poor at approximately 15 months (2). Moreover, GBM has high recurrence rates (>90%) compared to other cancer types (3, 4). In the recurrent setting, treatment options include reoperation, re-irradiation, and combined therapy (5). Imaging techniques and tissue biopsies are used to characterize the tumour and predict treatment response (6). Nevertheless, not all patients are eligible for operation and the resection or biopsy of the tumour may present risks, such as brain swelling or affect neurological functions (7).

In this context, interest in the use of liquid biopsies in GBM management is emerging (8, 9). Liquid biopsy is defined as the sampling and analysis of biomolecules in biofluids, such as blood, urine and saliva (10, 11). This approach aims to capture tumour activities in real-time to be used in the diagnosis and prediction of disease progression in a minimally invasive way. Tumoural content that can be shed into circulation includes circulating tumour DNA (ctDNA) and circulating tumour cells (CTCs). CTCs are cells that detach from the tumour and reach the bloodstream (10). This is thought to be due to a process that cells undergo called epithelial-mesenchymal transition (EMT) (12) in which cells downregulate epithelial markers and upregulate mesenchymal markers, gaining migratory and invasive properties. CTCs contribute to tumour metastasis and their detection may be used as a biomarker to predict disease outcome and monitor response to treatment (13). Recent literature has shown that GBM sheds tumoral content into the circulation (14, 15) and CTCs (16–18) can be detected in patients’ blood. In brain tumours, extracranial metastatic events are rare, possibly due to the presence of the BBB, short survival rate and suppression of brain cell growth extracranially by the immune system (16, 19). Nevertheless, detection of GBM cells in peripheral blood of patients can facilitate the assessment of tumoural information without the need for invasive approaches.

CTCs are rare events in circulation, with an average of 1-10 cells per 10 ml of blood depending on the cancer type (10). In GBM, Muller et al. reported CTC counts ranging from 1 to 22 cells per 2.1 × 10^6^ mononuclear cells (MNCs) (16). This highlights the challenge for current techniques to isolate cells in a sensitive and reproducible way for further characterization. Currently, the only FDA-approved platform for CTC isolations is the CellSearch^®^ system (Menarini Silicon Biosystems, Italy) (20, 21) which enumerates CTCs of epithelial origin and consists of a positive selection of EpCAM+ cells. Nevertheless, so far, this method has not been applied for the isolation of CTCs from GBM since these cells tend to present a more mesenchymal phenotype (17). This emphasizes the need for a different approach to be implemented to isolate CTCs in non-epithelial tumours. To overcome this limitation, label-free technologies are emerging as potential platforms to fulfil this need (22). These techniques explore the physical properties of tumour cells, as size and deformability, which tend to differ from white blood cells (WBCs) in circulation (23). Among these technologies, the spiral microfluidic device is an alternative device that sorts cells by size using a combination of inertial lift force and Dean drag forces (24). This technique allows relevant blood volumes to be processed rapidly for the enrichment of CTCs. This technology has been used to isolate CTCs from other solid epithelial cancer types such as head and neck cancer (25), lung cancer (26), breast cancer (27) and melanoma (28). However, there are no studies to date on the application of this spiral microfluid technology for the isolation of CTCs from GBM patients.

Another challenge faced in the field is the characterization of CTCs from GBM. Currently, no marker can specifically confirm GBM cell in origin. We have elected glial fibrillary acidic protein (GFAP) and cell-surface vimentin (CSV) as markers to differentiate putative GBM cells in circulation from other blood cells. GFAP has high sensitivity and specificity for cells of neural origin and primary brain cancer cells. It has previously been observed that GFAP-positive cells found in peripheral blood of GBM patients present the same genomic aberrations with matching tumour tissue by genomic hybridization, sequencing analysis and fluorescence in-situ hybridization (FISH) (16). In addition to GFAP, the marker CSV was included in our characterization. CSV is a mesenchymal CTC marker and is mostly associated with tumour cells (29, 30). CTCs from GBM were shown to have a mesenchymal phenotype (17) and CSV has been detected in GBM cancer stem cells (CSC) (31). Also, CSV+ CTCs enumeration has been shown to correlate with prostate cancer progression (32).

In this pilot study, we used a spiral microfluidic technology to isolate CTCs from peripheral blood of 20 newly diagnosed GBM patients, before and after surgery. We characterised these cells by immunofluorescence staining, using GFAP, CSV and DNA FISH for EGFR amplification. We detected CTCs in thirteen patients in total, including nine patients before surgery and eleven patients after surgery. Further analysis of patients’ clinical outcomes showed that patients with CTC count 0 after surgery presented significantly prolonged recurrence-free survival. This is the first study of its kind confirming CTC enrichment from whole blood of GBM patients using a spiral microfluidics chip.

## Material and Methods

### Cell Culture

Q-Cell primary GBM cell lines have been developed and characterised in detail, data is publicly available from Q-Cell https://www.qimrberghofer.edu.au/q-cell/ (33, 34). GBM lines are maintained as glioma neural stem cell (GNS) cultures (35) or as neurosphere cultures using StemPro NSC SFM (Invitrogen) as per manufacturer’s guidelines. U87MG and U251MG and Q-Cell patient-derived cell lines (BAH1, PB1 and MN1) were kindly gifted by Prof. Bryan Day (QIMR, Brisbane, Australia). All cells were cultured under standard conditions in humidified incubators at 37°C, 5% CO_2_. For U87MG and U251MG cell lines, RPMI-1640-Glutamax (Life Technologies, Inc) supplemented with 10% foetal bovine serum, FBS (Life Technologies, Inc) and 1% Penicillin-Streptomycin (Life Technologies, Inc) was used. Cell dissociation was done using TryPLe (Thermo Fisher Scientific, USA). For patient-derived cell lines (BAH1, proneural; MN1, mesenchymal; PB1, classical; HW1, classical) culture, flasks were previously coated with Matrigel (1:100 in PBS). Cells were maintained using StemPro NSC SFM media (Thermo Fischer) as per manufacturer’s instructions. This media contains KnockOut™ DMEM/F12, StemPro^®^ Neural Supplement, FGF-basic (AA 10–105) Recombinant Human, EGF Recombinant Human. Exclusively for BAH1 cells, no EGF supplementation was added to the media. Cell dissociation was done using Accutase^®^ solution (Merck) followed by its inactivation using Trypsin inhibitor (Merck). Cell lines were STR profiled for authenticity and were confirmed negative for mycoplasma infection by PCR.

### Spiking Experiments

GBM cell lines were labelled using Cell Tracker™ Green CMFDA (5-chloromethylfluorescein diacetate) (Life Technologies) as per manufacturer’s instructions. Subsequently, cells were observed under a fluorescence microscope to check if labelling was efficient. Cells were counted and different numbers of cells were spiked in healthy control whole blood or directly into white blood cells. The sample was loaded into a 10 ml syringe and pumped through the spiral chip at 1.7 ml/min. Both outlet tubes (CTC and waste) were connected to a falcon tube. Subsequently, total volume from both outputs was seeded in p96 well plates and counted manually using fluorescence microscopy (Nikon Eclipse Ts2). Recovery rates were defined as (Stained cells on CTC output)/(stained cells on CTC + stained cells on waste output) (25, 28).

### Patient Recruitment and Ethics

This study was approved by the human research ethics committee (HREC) of Royal Brisbane and Women’s Hospital (Brisbane Australia), approval number: HREC/2019/QRBW/48780, and the Queensland University of Technology (approval number: 1900000292). All documents were acknowledged by the RBWH research governance (RGO). Samples were collected between June 2019 and November 2020. All participant patients gave their written consent to participate in this study and blood samples were collected from patients before and after brain surgery or needle biopsy.

### Spiral Microfluidic Technology

Blood was collected in EDTA tubes and incubated with a red blood cell (RBC) lysis buffer (Astral Scientific). Cells were then centrifuged (500 x g for 10 min) and the pellet resuspended in 10 ml sheath buffer (PBS containing 2mM EDTA, 0.5% BSA). Samples were loaded into a 10 ml syringe and pumped through the spiral chip at 1.7 ml/min. Both outlet tubes (CTC and waste) were connected to a 15 ml falcon tube. Both outputs collected were spun down at 1200 RPM for 5 min, fixed using 4% PFA for 10 min. Subsequently, enriched cells were washed (PBS) three times and CTC output was immediately cytospun onto glass slides using the Cytospin™ 4 Cytocentrifuge (ThermoScientific, USA).

### Immunofluorescence

Following enrichment, slides were permeabilised using 0.1% Triton-X 100 for 10 min at room temperature (RT) and incubated with blocking solution (10% FBS) for 1 h at RT. Cells were stained with GFAP Polyclonal Antibody (Dako), cell surface vimentin (Abnova) and CD45 Monoclonal Antibody (Abcam) diluted in blocking solution. A summary of the staining conditions and antibody dilutions is shown in **Table S2**. Subsequently, slides were stained with DAPI (1:1000; stock solution 1mg/ml) for nuclear staining for one minute at RT. Slides were mounted using ProLong™ Gold Antifade (Invitrogen), coverslipped and imaged using a Zeiss Axio Imager Z2 microscope.

### Fluorescence In-Situ Hybridization (FISH)

DNA FISH for the detection of EGFR amplification was carried out using EGFR/CEN-7probes (SureFISH 7p11.2 EGFR 188kb RD; SureFISH Chr7 CEP GR - Agilent) according to the manufacturer’s protocol using a FISH accessory kit (Dako, K5799). This experiment was performed in the same slides in which putative CTCs were identified by IF. Briefly, the slides were incubated in pretreatment buffer at 98°C for 10 min; followed by incubations in wash buffer and incubation with pepsin. The slides were then incubated in ethanol series (70%, 85% and 90%) and the probe mix was incubated at 90°C for 5 min followed by overnight incubation at 37°C. Slides were counterstained with DAPI, coverslipped and imaged on a Zeiss Axio Imager Z2 microscope in FITC and texas red channels for detection of the chromosomal-7 and EGFR signals, respectively. The number of EGFR signals was compared to the centromeric probe to determine whether the signal was amplified.

### Statistical Analyses

All statistical analyses were performed using JMP Pro version 15.2.1 (SAS Institute, Cary, NC, USA). CTC numbers are reported as whole numbers in 1 ml of whole blood. CTC counts before and after were compared to patients’ outcomes using a negative binomial distribution. In addition, recurrence-free survival curves were estimated by Kaplan Meier analyses which were based on the number of CTCs (patients with CTC ≥1 and CTC = 0) before or after surgery and the clinical outcome. The time to disease progression or death was calculated by the time elapsed between the blood collection date and the date of clinical progression, death, or the last follow-up visit with an MRI scan image. Differences were considered statistically significant when *p* ≤ 0.05.

## Results

### Recovery Rates Using GBM Cell Lines

**Figure 1:**
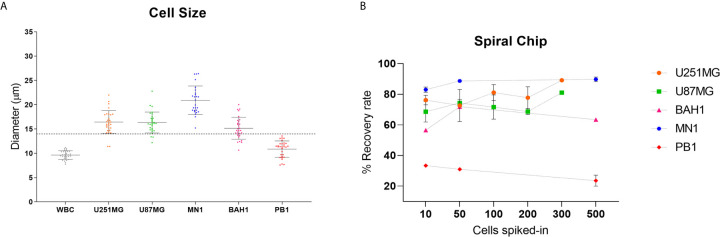
Recovery studies using GBM cell lines. **(A)** Cell size distribution of five different GBM cell lines (U251MG, U87MG, MN1, BAH1 and PB1) and white blood cells (WBCs). Cells were harvested and plated into a 96 well plate. Cell diameter was measured using Nikon Eclipse Ti-S. Software NIS-Elements, n = 30. The dashed line represents the cutoff of the spiral microfluidic device used (14 µm). **(B)** Spiral Chip recovery rates. Different numbers of GBM cell lines labelled with a Cell Tracker™ Green CMFDA (5-chloromethylfluorescein diacetate) (Life Technologies, USA) were spiked into whole blood and/or directly into WBCs. Samples were pumped through the spiral chip at 1.7ml/min. All volume was distributed into a 96 well plate and counted using fluorescence microscopy (Nikon Eclipse Ts2). Recovery rates were calculated as the total of labelled cells found in the CTC-output/total of labelled cells from both outputs. For U87MG and U251MG recovery rates using 10, 50, 100, 200 cells, n = 3. For 300 cells, n = 1. For MN1, BAH1 and PB1 recovery rates using 500 cells and 10 cells for MN1, n = 2. For recovery rates using 10 and 50 cells (BAH1, PB1), n = 1. Error bars indicate the standard deviation from the mean value across replicates.

In order to optimise the spiral microfluidic device, two immortalized GBM cell lines (U251MG, U87MG) and three primary Q-Cell GBM cell lines (BAH1, MN1, PB1) were used (33, 34, 36). This panel of cell lines exhibited different cell sizes, to assess the efficiency of detection using the microfluidic chip (**Figure 1A**). We next labelled cells with a Cell Tracker™ Green CMFDA (5-chloromethylfluorescein diacetate) (Life Technologies, USA) and spiked GBM cells into whole blood from healthy controls or directly into white blood cells diluted in PBS (~1x10^6^ cells/ml). Different cell quantities were spiked-in (10, 50, 100, 200, 300 and 500) in the attempt to reflect clinically relevant numbers. Both U251MG and U87MG cell lines presented similar recovery rates, ranging from 60 to 80% (**Figure 1B**), as expected from the comparable cell diameter size. When patient-derived cell lines (MN1, BAH1 and PB1) were assessed, recovery rates differed from 23 to 90%, according to their size (**Figure 1B**). MN1 has the largest cell size, presented the highest recovery rates (approximately 90%). BAH1 recovery rates ranged from 56 to 71% whereas the smallest cell line, PB1, had the lowest recovery rates from 23 to 33%.

### Patients Cohort and Collection Timepoints

**Figure 2:**
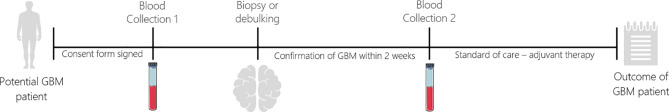
Schematic overview of timepoints for blood collections in potential GBM patients.

A total of 20 GBM patients and 3 healthy controls (HC) have been investigated in this study. Blood from GBM patients was collected before and after surgery. A schematic overview of timepoints for blood collections in potential GBM patients is shown in **Figure 2**. The average age for GBM patients was 60.7 years (ranging from 37 to 82) whereas for HC was 31 years (ranging from 27 to 35). The clinicopathological findings of GBM patients are presented in **Table 1**. The majority of patients in this study were IDH wildtype, presented with variable p53 staining and were positive for ATRX. MGMT status information was not available (**Table 1**).

**Table 1:** Demographic and clinical information of GBM patients.

Characteristic	n	% of total
Total patients enrolled	20	
Gender		
Female	10	50
Male	10	50
Age		
30-40	1	5
40-50	2	10
50-60	6	30
60-70	8	40
70-80	2	10
80-90	1	5
P53 (positive)		
Majority positive (>50%)	5	25
Variable positive (≤50%)	10	50
% not available	5	25
ATRX		
Positive	18	90
Negative	1	5
Not available	1	5
IDH-1-R132H		
Positive	1	5
Negative	18	90
Not available	1	5
Ki67		
Increased	6	30
Not available	14	70
MGMT status		
Not available	20	

### Enrichment and Characterization of Putative CTCs From GBM Patients

**Figure 3:**
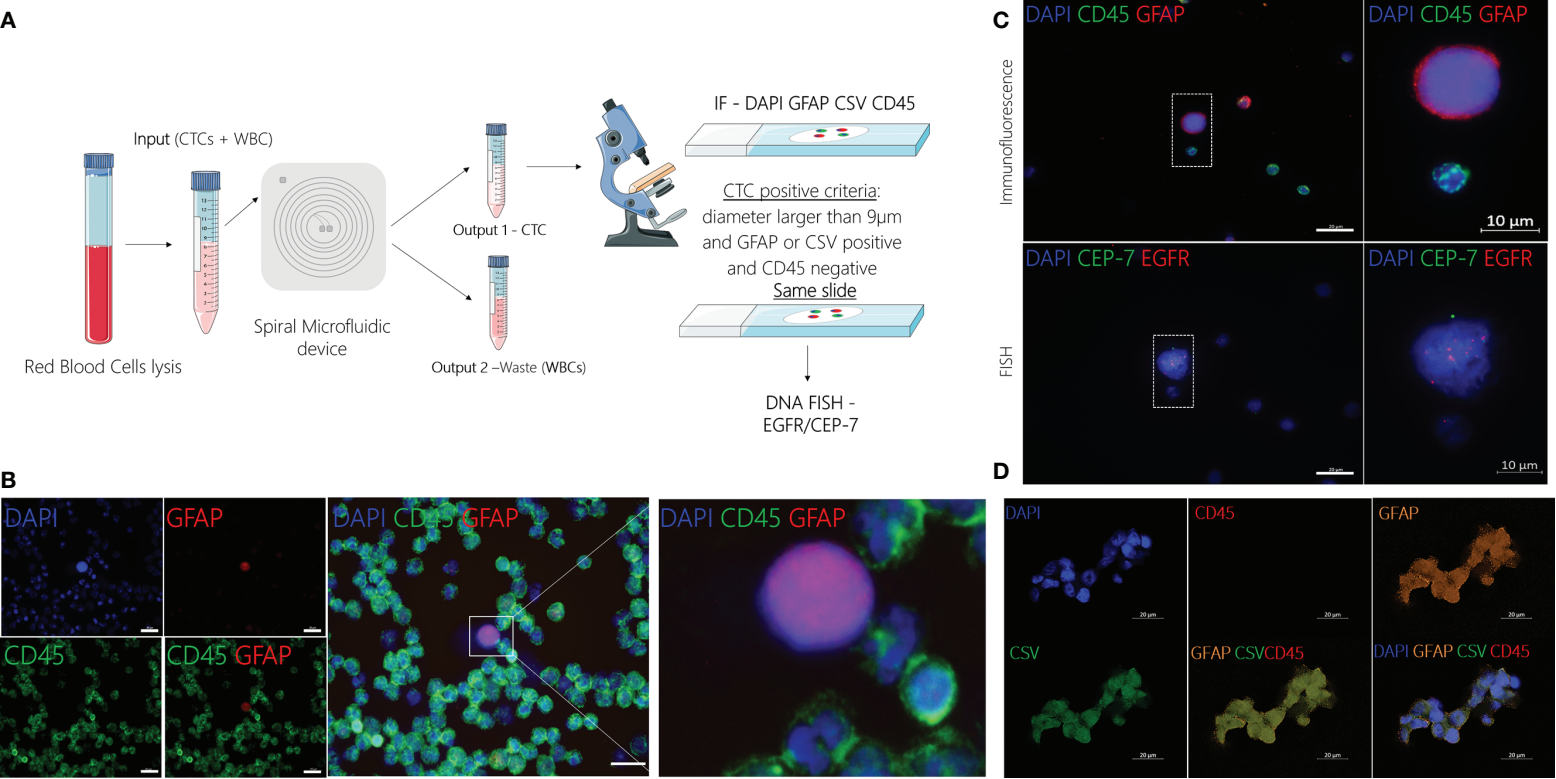
**(A)** Schematic representation of CTC isolation using the spiral microfluidic device and characterization using IF (GFAP/CSV/CD45) and FISH (EGFR amplification). **(B)** Representative image of CTC characterization using immunofluorescence targeting GFAP (red), CD45 (green) and DAPI (blue), scale bar = 20µm. **(C)** Characterization of putative CTC at a molecular level using DNA FISH to detect EGFR (red) copies and CEP-7 (green). **(D)** Characterization of putative CTC cluster using immunofluorescence targeting GFAP (orange), CSV (green), CD45 (red) and DAPI (blue), scale bar = 20µm.

GBM patients’ blood was collected in two different time points, before and after surgery (**Figure 2**). After collection, blood samples from patients and healthy controls were processed within two hours using the spiral microfluidic device (**Figure 3A**). Enriched cells were characterized using immunofluorescence, targeting GFAP, CSV and leukocyte common antigen (CD45) (**Figure 3B**). CD45 staining was used to exclude white blood cells. Putative CTCs were considered positive when met the criteria of (1) cell diameter of at least 9µm and (2) DAPI positive (nuclei staining), GFAP or CSV positive and CD45 negative staining (16). No GFAP+ or CSV+/CD45- cells were found in peripheral blood of the control group. In GBM patients before surgery, CTCs were found in 9 out of 20 patients (45%), whereas after surgery in 11 out of 19 patients (58%). Cell counts varied between 1 to 24 cells per ml of whole blood. In addition to single CTCs, cell clusters were observed (**Figure 3D**) in two different samples.

After immunofluorescence, characterization at the molecular level was performed in a subset (n=3) of samples using DNA FISH to detect EGFR amplification. EGFR signal was measured in the nuclear area and the number of EGFR probe to chromosome 7 centromere probe was measured per cell. In cells where EGFR probe was found to be 3 or more times higher than the chromosome 7 centromere probe, this was recorded as an amplification (**Figure 3C**). CTC counts for all GBM patients, including the time of collection and EGFR amplification results are shown in **Table S1**.

### CTC Counts and Clinical Data

**Figure 4:**
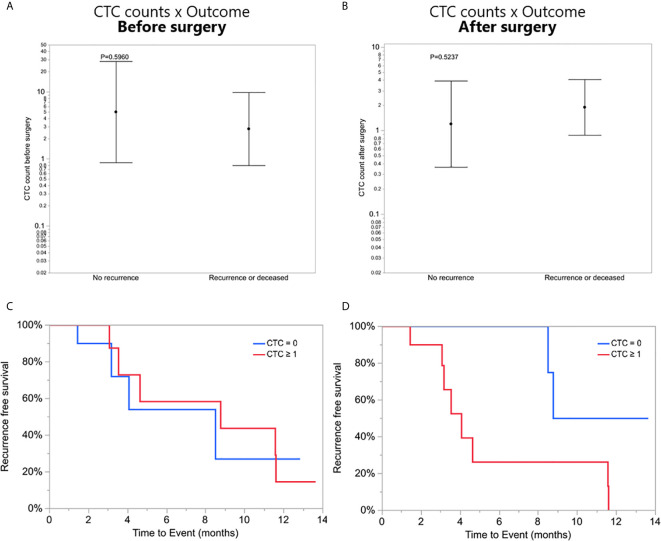
**(A, B)** CTC counts of patients with poor outcomes (recurrence or death) versus patients with good outcomes (stable disease and no recurrence), before and after surgery, respectively. **(C, D)** Recurrence-free survival. Kaplan-Meier curves showing recurrence-free mean survival time of CTC = 0 group and CTC ≥1 group before and after surgery, respectively.

To investigate a potential clinical utility of the presence or enumeration of CTCs in the blood of GBM patients, CTCs counts were coupled with clinical data from patients (**Table 2**). The outcome of patients has been evaluated after 17 months from the collection of the first patient. The time to disease progression or death was calculated by the time elapsed between the collection date and the date of clinical outcome. The recurrence-free survival curves were estimated by Kaplan Meier analysis for patients with (≥1) and without CTCs (=0), before (**Figure 4C**), and after surgery (**Figure 4D**) (log-rank p=0.7705, 0.0370, respectively). Interestingly, patients with a CTC count equal to 0 after surgery presented with significantly prolonged recurrence-free survival curves compared to patients with ≥1 CTCs. Also, CTC counts of patients with poor outcomes (recurrence or death) versus patients with good outcomes (stable disease and no recurrence) were compared using a negative binomial distribution. Nevertheless, there was no statistically significant association between CTC counts and patients’ outcomes before (p=0.5960) (**Figure 4A**) or after surgery (p=0.5237) (**Figure 4B**). Patients with CTC >= 1 were more likely to have recurrence (80%) versus patients with CTC = 0 (20%) but the small number of patients means this difference was not statistically significant (Likelihood Ratio chi-square test, p=0.1247).

**Table 2:** CTCs counts and clinical data from GBM patients.

Pt	Tumour location	Tumour volume (cm3)	Oedema*	Enhancement*	Necrosis*	Extent of resection*	CTC count Before/after surgery		Outcome
01	Left side brain	20.0	++	ring	++	Biopsy	1	2	Deceased
02	Right temporal lobe	14.4	+	confluent	+	Near total	1	3	Deceased
03	Left frontal	1.7	+	ring	++	Complete	1	0	No recurrence
04	Right parietal	26.3	+++	ring	+	Complete	0	0	No recurrence
05	Left temporal	38.7	+++	confluent	+	Near total	17#	3#	Recurrence
06	Right temporal	83.2	+	ring	++	Near total	5	5	Deceased
07	Right parietal brain lesion	24.8	+++	confluent	+	Debulking	0	0	Recurrence
08	Left frontal lesion	4.4	++	confluent	+	Near total	1	0	Deceased
09	Right side brain lesion - frontal	53.8	+++	ring	++	Debulking	0	2	Deceased
10	Right frontal lesion	44.9	+++	ring	++	Near total	3	2	Deceased
11	Right frontal lesion	40.5	+	ring	++	Biopsy	0	3	No recurrence
12	Temporal tumour	33.0	++	thick	+	Near total	0	1	Recurrence
13	Right temporal lobe	39.9	+++	thick	++	Complete	0	0	No recurrence	
14	Right parietal	63.8	++	ring	++	Near total	0	1	Recurrence
15	Right temporal tumour	39.5	++	Ring	++	Complete resection	24#	3	Deceased
16	Left frontotemporal	14.3	+++	Ring	++	Complete resection	0	0	N/A
17	Bifrontal and corpus callosal	50.0	+	Thick	++	Biopsy	0	N/A	N/A
18	Left frontal	19.0	++	Ring	++	Debulking	0	0	N/A
19	Right frontal	30.2	+++	Ring	++	Debulking	2	1	N/A
20	Right frontal	57.6	–	Confluent	–	Complete	0	0	N/A

*Oedema on FLAIR; – no significant oedema; + diameter of oedema less than tumour; ++ diameter of oedema similar to tumour; +++ diameter of oedema greater than tumour.

*Enhancement: - Ring: periphery only: typical thin ring-enhancing, confluent, including thick ring enhancement).

*Necrosis: + necrosis present; < half the diameter; ++ necrosis; > half diameter.

*Resection: biopsy, debulking, near total resection ie >0.175 cm2 (one voxel) but < 1cc of residual tumour, complete (no residual enhancement).

*N/A, not available; #, including CTC clusters.

## Discussion

One of the challenges in the CTCs field pertains to the isolation and enrichment technologies as CTCs are very rare events in blood. Sensitive techniques which can reliably and reproductively isolate CTCs are currently lacking. There are few studies on the detection of CTCs in patients with GBM using different approaches for isolation and characterization and also different cohorts, including newly diagnosed patients, or patients with progressive or stable disease (16–18, 37–40). For example, Muller et al. analysed newly diagnosed and also recurrent patients, using density-gradient centrifugation and considered as putative CTCs, cells that were GFAP positive, whereas Sullivan et al. used the CTC-iChip and considered as CTCs cells that were positive for SOX2 or Tubulin beta-3 or EGFR or A2B5, and c-MET. MacArthur et al. used an improved version of the isolation technique used by Muller et al., nevertheless, the use of density-gradient centrifugation methods may result in low enrichment and purity whereas microfluidics methods could result in higher purities (41, 42). In our study, we assessed the spiral microfluidic device for CTC isolation. This technology presents some advantages such as the ability to process large volumes of blood, easy manipulation, speed and cost-effectiveness (24, 25). This technology is also capable of isolating CTC clusters which are known to increase the metastatic potential when compared to single CTCs (43). CTC clusters were recently reported in GBM (39), nevertheless, further research should be carried out to establish a clinical significance. Cells isolated using the spiral chip are still viable and could be used for downstream analysis. However, when only few cells are found, an extended profile analysis may not be possible. Another drawback of this technology is that this chip sorts cells by size, and cells that are smaller than 14 µm would be lost. In addition, after enrichment through the spiral microfluidic device, we transfer the cell pellet onto glass slides and this step may also include bias.

Five cell lines, including patient-derived cell lines covering all GBM molecular subtypes, were used to optimise the spiral device and to assess recovery rates of captured cells. The average cell size varies (from 11 to 21 µm) among all cell lines, larger variation was seen in the patient-derived cell lines. This finding reflects that the tumour cells are heterogeneous, known to be a hallmark of GBM (44). In addition, the WBCs diameter was also measured, with a mean size of approximately 10 µm. WBCs consist of different cell types which have varied diameters, lymphocytes (small 7-8 µm; large 12-18 µm), neutrophils (9-15 µm), eosinophils (9-15 µm), basophils (10-16 µm) and monocytes (12-20 µm) (45). Therefore, WBCs’ mean diameter may range from ~10 to 15 µm (24) which is consistent with our finding. Since the majority of GBM cell lines had diameters larger than the cut-off of the chip and were successfully sorted, these results encouraged us to proceed using the spiral microfluidic technology for GBM clinical samples for CTC enrichment.

In our study, blood samples from newly diagnosed GBM patients were collected before (n = 20) and after surgery (n = 19) and CTC numbers were assessed. After isolation with the spiral microfluidic device, putative CTCs were characterized at the protein level using immunofluorescence. Currently, there is no specific marker to characterise GBM cells. Our analysis included GFAP, CSV and CD45 to exclude white blood cells. GFAP is an intermediate filament protein which is strongly expressed in mature astrocytes (46) and has previously been used to characterize putative CTCs found in peripheral blood of GBM patients (16). Vimentin is also an intermediate filament protein which is associated with cellular motility and is upregulated in CTCs derived from GBM patients (17). In addition to vimentin’s intracellular functions, vimentin can be recruited to the cell surface (cell-surface vimentin - CSV) and contribute to different cell processes, like migration, adhesion, and cell signalling. Interestingly, CSV is mostly expressed in tumour cells (29) and has been used as a CTC mesenchymal marker in other cancer types (29, 30, 32). Our conservative CTC-positive criteria included cells that were larger than 9 µm, positive for DAPI, positive for GFAP or CSV and negative for CD45.

CTCs were found in peripheral blood of thirteen out of the twenty patients analysed (65%). However, no cells matching the CTC criteria were found in blood of healthy controls. In nine of the patients, cells were detected before surgery (45%) and in eleven of them, after surgery (58%). These findings present comparable rates to that of other studies (17, 18, 37). Sullivan et al. reported CTCs in 13 out of 33 patients (39%), MacArthur et al. found CTCs in 8 out of 11 patients (72%), whereas Gao et al. detected CTCs in 24 out of 31 patients (77%). Cell counts varied from 1 to 24 cells per ml of whole blood, similar to other studies (16). Nevertheless, taking into consideration our processing method limitation, these numbers could be higher. In addition to single CTCs, putative CTC clusters have been detected (**Figure 3D**). The presence of CTC clusters in GBM patients has been reported for the first time by Krol et al. in 2018 (39). In the study, the authors used Parsortix cassettes to capture CTCs and identified clusters in seven out of 13 patients (53.8%) from a clinical trial with recurrent or progressive GBM. In the Parsortix system, captured cells (“CTCs”) are caught in the Parsortix filtration cassette due to their larger size and lower compressibility than other blood components and cells of interest are kept in the cassette. This technology differs from the one used in the present study, the spiral microfluidics, in which larger cells (“CTCs”) focus near the inner wall due to the combination of the inertial lift force and the Dean drag force at the outlet WBCs would be trapped inside the core of the Dean vortex formed closer to the outer wall. Therefore, the cells of interest run through the chip and are found in an external tube connected to the spiral device. In our study, we observed CTC clusters in three different samples from 2 out of 20 (10%) with newly diagnosed GBM patients. Both of these patients (#05 and # 15, **Table 2**) presented a poor outcome (recurrence or deceased). These interesting findings suggest the ability of these circulating cells to cross the blood-brain barrier but larger studies are required to test the reproducibility of these data and potential clinical value in GBM patients.

Further characterization was carried on assessing EGFR amplification. EGFR is one of the most frequently mutated genes and amplification or overexpression is present in around >50% of the cases (47, 48). Also, Muller et al. reported an association between EGFR amplification and CTCs release, suggesting EGFR signalling may have a role in supporting the dissemination of GBM (16). In the present study, due to the technique used (cells of interest being fixed onto slides for characterization), in addition to the small CTC counts in the majority of patients, we were not able to perform further characterization on these cells.

To explore the clinical value of CTCs in GBM patients, the recurrence-free survival time of patients with CTC = 0 and patients with CTC ≥1 before and after surgery was assessed. This analysis showed that patients that had CTC counts after surgery equal to 0, had significantly longer recurrence-free survival time (p = 0.0370) compared to patients with 1 or more CTCs. Due to the limitation of a small cohort in this study, it is not yet possible to confirm the clinical value of CTCs in GBM. Nevertheless, these results encourage more studies to investigate the clinical significance of CTCs in GBM patients.

## Conclusion

This is the first study using the spiral microfluidic device for the enrichment of CTCs found from peripheral blood of newly diagnosed GBM patients. We isolated putative CTCs in a label-free and cost-effective way from 13 out of 20 patients, before or after surgery. These putative cells were then characterized using IF (GFAP, CSV, CD45, DAPI) and FISH (EGFR amplification). This is a pilot study and has limitations such as a small cohort and the lack of specific markers for the characterization of CTCs from GBM. However, our results reinforce the knowledge that GBM can shed content into circulation and highlight the importance of studies using the spiral microfluidic technology - an easy, fast and cost-effective technique for CTCs enrichment. Further research should be undertaken to investigate the clinical value of CTCs in GBM patients, and our results encourage the use of this label-free technique to improve this understanding in the future.

## Data Availability Statement

The original contributions presented in the study are included in the article/**Supplementary Material**. Further inquiries can be directed to the corresponding author.

## Ethics Statement

This study was approved by the human research ethics committee (HREC) of Royal Brisbane and Women’s Hospital (Brisbane Australia), approval number: HREC/2019/QRBW/48780, and the Queensland University of Technology (approval number: 1900000292). All documents were acknowledged by the RBWH research governance (RGO). All participant patients gave their written consent to participate in this study and blood samples were collected from patients before and after brain surgery or needle biopsy. The patients/participants provided their written informed consent to participate in this study.

## Author Contributions

Study design: JB, AK, BC, BD, RJ, and CP. Experimentation: JB and AK. Data analysis: JB, AK, GH, PL, MW, BC, BD, RJ, and CP. Manuscript preparation and review: all authors. All authors contributed to the article and approved the submitted version.

## Funding

JB is funded by ATM LATAM QUT Postgraduate Research Scholarship. CP is currently receiving funding from the National Health and Medical Research Council (APP 2002576), Cancer Australia (APP1145657) and the Garnett Passé and Rodney Williams Foundation. BD received funding from The Sid Faithfull Group and Cure Brain Cancer Foundation to conduct this study.

## Conflict of Interest

The authors declare that the research was conducted in the absence of any commercial or financial relationships that could be construed as a potential conflict of interest.
