# Supplementary material for: Isolation of Circulating Tumour Cells in Patients With Glioblastoma Using Spiral Microfluidic Technology – A Pilot Study
Source: Front Oncol. 2021 Jun 3;11:681130. doi: 10.3389/fonc.2021.681130 (PMC8210776; doi:10.3389/fonc.2021.681130)
Supplement: Supplementary file 1 [file DataSheet_1.pdf]

Supplementary Material

Table S1. CTC counts of 20 GBM patients (before and after surgery)

	**CTC (spiral/ml of blood)**	**Marker**	**Collection**	**CTC (spiral/ml of blood)**	**Marker**	**Collection**
	**Before surgery**			**After surgery**		
1	1	GFAP*	2 days before surgery	2	GFAP	5 days after surgery
2	1	GFAP	3 days before surgery	3	GFAP	4 days after surgery
3	1	GFAP	Same day	0	-	3 days after surgery
4	0	-	1 day before surgery	0	-	3 days after surgery
5	17[#]	GFAP CSV	Same day	3[#]	GFAP CSV*	9 days after surgery
6	5	GFAP*	1 day before surgery	5	GFAP	2 days after surgery
7	0	-	Same day	0	-	3 days after surgery
8	1	GFAP	1 day before surgery	0	-	3 days after surgery
9	0	-	3 days before surgery	2	GFAP	10 days after surgery
10	3	GFAP	1 day before surgery	2	GFAP	7 days after surgery
11	0	-	Same day	3	GFAP	5 days after surgery

12	0	-	Same day	1	GFAP<br>CSV	6 days after surgery
13	0	-	1 day before surgery	0	-	5 days after surgery
14	0	-	1 day before surgery	1	CSV	6 days after surgery
15	24#	GFAP	Same day	3	GFAP<br>CSV	2 days after surgery
16	0	-	3 days before surgery	0	-	4 days after surgery
17	0	-	Same day	N/A	-	-
18	0	-	Same day	0	-	3 days after surgery
19	2	GFAP<br>CSV	Same day	1	GFAP	5 days after surgery
20	0	-	Same day	0	-	5 days after surgery

* = EGFR amplified; N/A = not available; # = cluster found

Table S2. Antibodies used and staining conditions

**Primary antibody**	**Dilution and incubation time**	**Secondary antibody**	**Concentration and incubation time**	**Isotype Control (same concentration and incubation time)**
**Anti-Glial Fibrillary Acidic Protein (GFAP) unconjugated. Dako –Z0334**	1:500<br>3 h - RT	Goat Anti-Rabbit IgG (Alexa Fluor 647) Abcam – ab150079 or<br>Goat Anti-Rabbit IgG (Alexa Fluor	1:1000<br>1 h -RT<br>Or	Rabbit IgG Isotype Control<br>Abcam - ab172730

		555) Abcam – ab ab150078)	1:2000<br>1 h -RT	
**Anti-CD45 (Alexa Fluor 488) Abcam – ab197730**	1:200<br>1:30h - RT	-	-	Mouse IgG2a Isotype control (PPV-04), Alexa Fluor 488 Thermo Fischer Scientific – MA5-18169
**Anti-CD45 – (APC) BD**<br>**340943**	1:50<br>1h - RT	-		Mouse IgG1, ϰ, Isotype Control (APC) BD 554681
**Cell Surface Vimentin (FTIC) - Abnova DH0043**	1:150<br>1h - RT	-		Mouse IgG2b, ϰ Isotype control (FITC) Biolegend 400309

A)

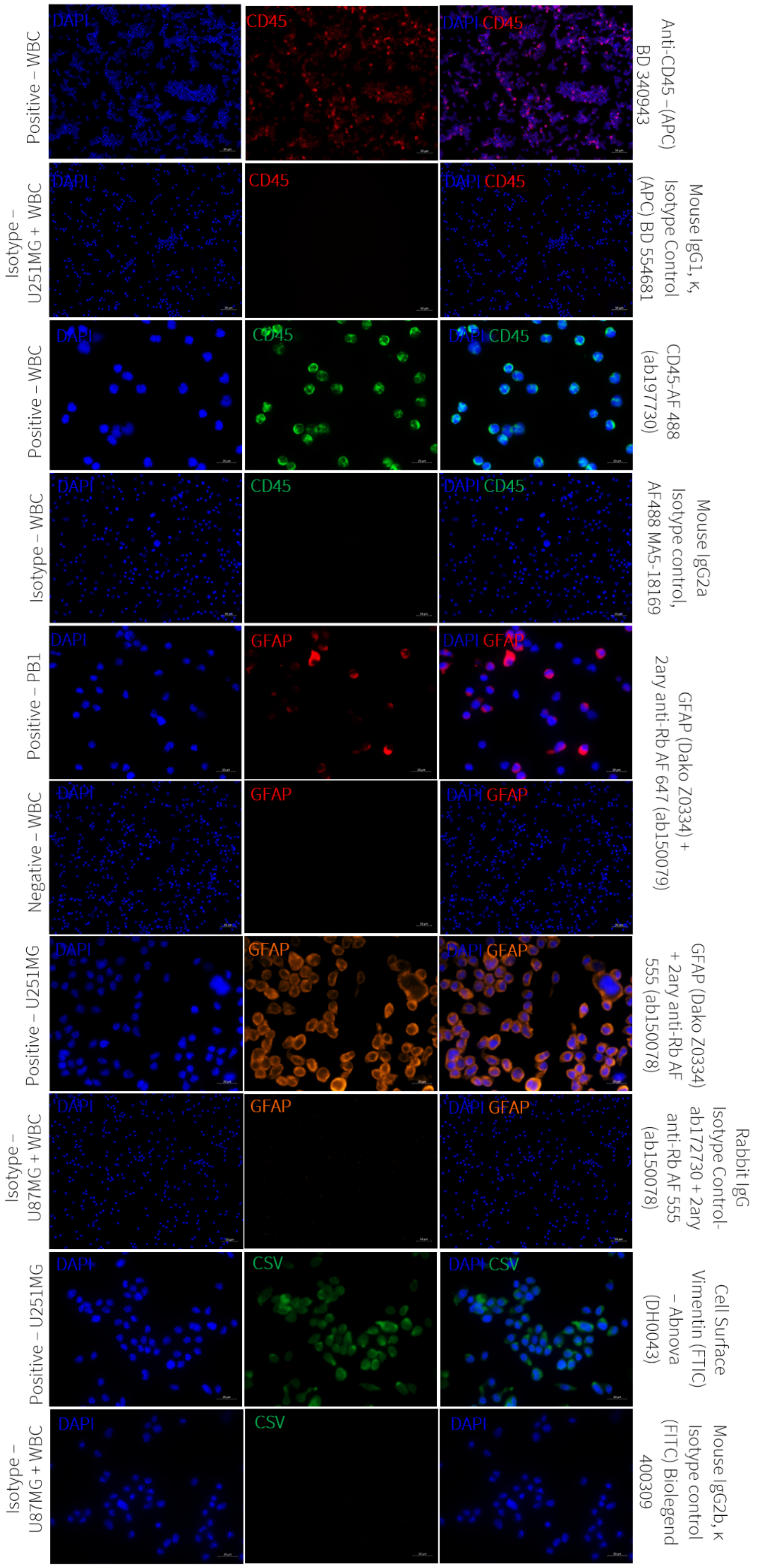

Positive – WBC
DAPI
CD45
DAPI CD45
Anti-CD45 – (APC) BD 340943
Isotype – U251MG + WBC
DAPI
CD45
DAPI CD45
Mouse IgG1, κ, Isotype Control (APC) BD 554681
Positive – WBC
DAPI
CD45
DAPI CD45
CD45-AF 488 (ab197730)
Isotype – WBC
DAPI
CD45
DAPI CD45
Mouse IgG2a Isotype control, AF488 MA5-18169
Positive – PB1
DAPI
GFAP
DAPI GFAP
GFAP (Dako Z0334) + 2ary anti-Rb AF 647 (ab150079)
Negative – WBC
DAPI
GFAP
DAPI GFAP
Positive – U251MG
DAPI
GFAP
DAPI GFAP
GFAP (Dako Z0334) + 2ary anti-Rb AF 555 (ab150078)
Isotype – U87MG + WBC
DAPI
GFAP
DAPI GFAP
Rabbit IgG Isotype Control- ab172730 + 2ary anti-Rb AF 555 (ab150078)
Positive – U251MG
DAPI
CSV
DAPI CSV
Cell Surface Vimentin (FTIC) – Abnova (DH0043)
Isotype – U87MG + WBC
DAPI
CSV
DAPI
Mouse IgG2b, κ Isotype control (FITC) Biolegend 400309

B)

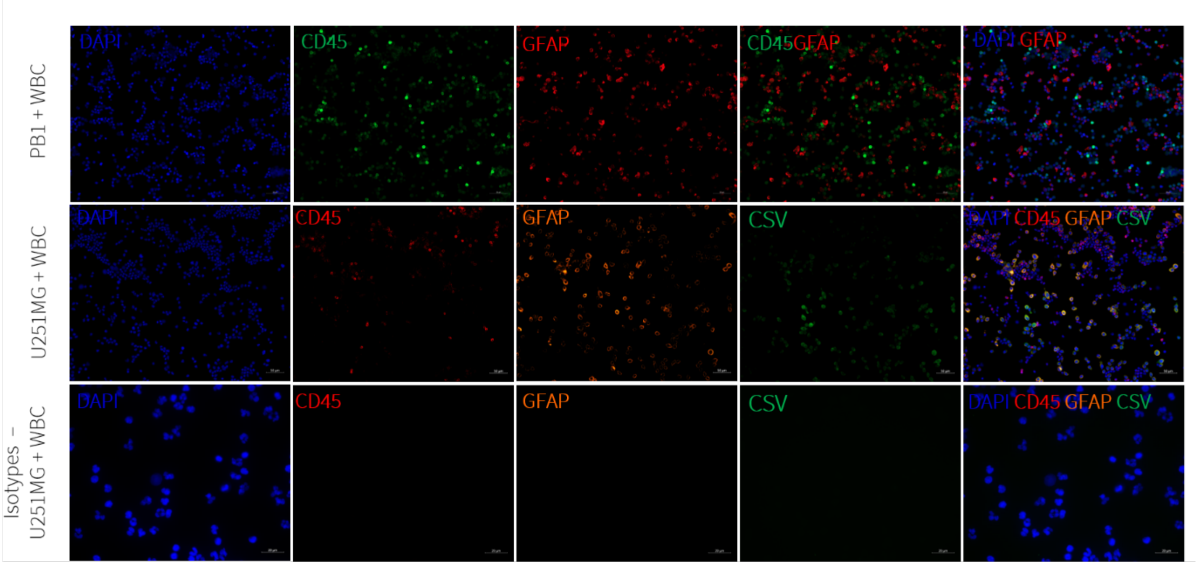

Figure S1. Validation panel of antibody. A panel of representative images consisting of negative and positive controls for all antibodies used in this study individually (A) and in combination (B).
